# Aza-heterocyclic Receptors for Direct Electron Transfer Hemoglobin Biosensor

**DOI:** 10.1038/srep42031

**Published:** 2017-02-07

**Authors:** Vinay Kumar, D. M. Nikhila Kashyap, Suraj Hebbar, R. Swetha, Sujay Prasad, T. Kamala, S. S. Srikanta, P. R. Krishnaswamy, Navakanta Bhat

**Affiliations:** 1 Centre for Nano Science and Engineering, Indian Institute of Science, Bangalore-560012, India; 2 PathShodh Healthcare Pvt Ltd, Bangalore-560012, India; 3 Anand Diagnostics Laboratory, Bangalore, India; 4 Samatvam Diabetes Endocrinology Centre, Bangalore, India

## Abstract

Direct Electron Transfer biosensors, facilitating direct communication between the biomolecule of interest and electrode surface, are preferable compared to enzymatic and mediator based sensors. Although hemoglobin (Hb) contains four redox active iron centres, direct detection is not possible due to inaccessibility of iron centres and formation of dimers, blocking electron transfer. Through the coordination of iron with aza-heterocyclic receptors - pyridine and imidazole - we report a cost effective, highly sensitive and simple electrochemical Hb sensor using cyclic voltammetry and chronoamperometry. The receptor can be either in the form of liquid micro-droplet mixed with blood or dry chemistry embedded in paper membrane on top of screen printed carbon electrodes. We demonstrate excellent linearity and robustness against interference using clinical samples. A truly point of care technology is demonstrated by integrating disposable test strips with handheld reader, enabling finger prick to result in less than a minute.

The point-of-care (POC) glucometer is an excellent example of how a simple and elegant idea can impact millions of lives. Clark and Lyons, through their seminal publication on glucose oxidase electrodes, laid the foundation for modern POC glucose sensors^1^. While most of the commercial glucose sensors are second generation, mediator assisted sensors, there has been substantial research in the recent past to enable third generation glucose sensors, by facilitating direct electron transfer between glucose oxidase enzyme and the working electrode^2^,^3^,^4^. Although the research publications in the field of biosensors have grown exponentially, in the last five decades, the commercialization has alarmingly lagged behind^5^,^6^,^7^,^8^,^9^. Many critical reviews and editorials have stressed the need for new strategies for biomolecule recognition, with an emphasis on novel receptor design for robust and reliable sensing^5^,^6^,^7^,^8^,^9^.

The receptor in the biosensor should be specific to the biomolecule of interest in the pool of bio-fluid and should be capable of giving a signal through an appropriate transduction mechanism. Antibodies and enzymes have become the first choice as a receptor molecule in POC biosensors^10^,^11^. While these biological receptors are highly sensitive and selective, when used with biochemistry analyzers in a pathology laboratory, their use in POC biosensors has significant limitations. By definition, a biosensor is a self-contained device^12^ that can be used in POC settings without any special storage and handling requirements. The stability of antibodies and enzymes, after functionalization on disposable strips and cartridges, is still a serious problem in POC biosensors. Moreover, the accuracy is also affected by ambient temperature, humidity and pH variations^10^,^13^.

A low cost and robust POC Hb biosensor can have a big impact on public health for the diagnosis of anemia, especially in underdeveloped countries of the world. There can be different reasons for anemia which include iron deficiency, B_12_ deficiency, nutritional conditions or other chronic conditions such as chronic kidney disease. According to WHO guidelines, Hb as a single biomarker, can be used for the diagnosis of anemia^14^. Hb testing is also very important during pregnancy, since it has been associated with an increased risk of preterm delivery^15^. The WHO Global database on anaemia for 1993–2005, covering almost half the world’s population, estimated the prevalence of anaemia at 25% worldwide while the prevalence is 43% in underdevelopment countries^16^,^17^. Anaemia affects 1.62 billion people globally with about 293 million children of preschool age, 56 million pregnant women and 468 million non-pregnant women estimated to be anaemic. Africa and Asia account for more than 85% of the absolute anaemia burden in high-risk groups. In India, almost 58% of pregnant women are anaemic and it is estimated that anaemia is the underlying cause for 20–40% of maternal deaths. India contributes to about 80% of the maternal deaths due to anaemia in South Asia^18^.

Hb testing was one of the oldest test done in pathology labs, and has evolved in the last several decades. Sahli’s acid hematin technique, Hoppe-Seyler’s carbon monoxide hemoglobin technique, Lovibond-Drabkin’s cyanmethemoglobin technique, Cyanide free SLS technique used in most of the hematology analyzers, are all based on colorimetric principle^19^,^20^,^21^,^22^. Hb has attracted biosensor research community, for the past several years^23^,^24^. Due to the presence of iron redox centre in the heme prosthetic group, electrochemical transduction seems to be a natural choice. Although Hb contains four iron (Fe^+2^) atoms in its structure, the iron center is embedded deep inside the globin chains and it is very difficult for the iron centre to communicate with the electrode surface for any practical sensing applications. Mediators such as ferricyanide, methylene blue, methylene green^25^,^26^,^27^, have also been employed for Hb biosensor. Ferricyanide, a redox active molecule, is an oxidizing agent for Hb and converts hemoglobin into methemoglobin. Hb is electrochemically assayed using ferricyanide as a mediator between hemoglobin molecule and electrode surface. The current at the electrode resulting from the oxidation of ferrocyanide to ferricyanide would be proportional to the concentration of Hb. Saithip Pakapongpan *et al*. described the electrochemical Hb biosensor based on methylene blue and carbon nanotube. Methylene blue is used as a mediator for Hb detection, where redox current of methylene blue depends on Hb concentration^26^. Christopher *et al*. proposed electrochemical Hb biosensor based on Poly (methylene blue) as a redox mediator^22^. Optical techniques such as surface plasmon resonance (SPR) have also been used for the detection of hemoglobin concentration in human blood^28^. The direct electron transfer of Hb embedded in novel electrode materials such as silver nanoparticles, dendrimers, graphene has been used to sense H_2_O_2_ and NO^29^,^30^,^31^. However, mediator free Hb biosensor, utilizing the direct electron transfer with heme centre has still been elusive.

## Results and Discussions

**Figure 1:**
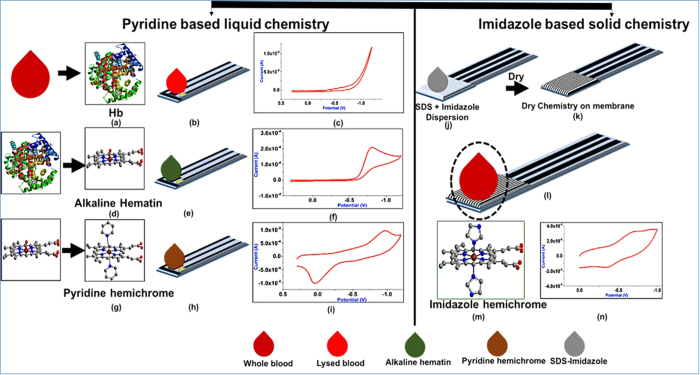
Pyridine and imidazole based concept for electrochemical detection of Hb. (**a**) Hb after lysing. (**b**) 300 uL lysed blood on carbon printed electrode. (**c**) CV of lysed blood. (**d**) Structure of Alkaline hematin. (**e**) 300 uL of alkaline hematin on carbon printed electrode. (**f**) CV of alkaline hematin. (**g**) Pyridine hemichrome. (**h**) 300 uL of pyridine hemichrome on carbon printed electrode. (**i**) CV of pyridine hemichrome. (**j**) dispersion of imidazole based chemistry on membrane laminated electrode. (**k**) Ready to use electrode with dried chemistry. (**l**) Imidazole-metHb hemichrome. (**m**) CV of imidazole hemichrome.

Herein, we demonstrate a novel analytical method for POC electrochemical detection of metalloproteins such as Hb. We propose new receptors based on aza heterocyclic compounds - pyridine and imidazole – to facilitate direct electron transfer between iron centres of Hb and working electrode. Figure 1 illustrates the overall sensing scheme using pyridine based liquid chemistry (a though i) and imidazole based dry chemistry (j through n). Figure 1(c,f) show the cyclic voltamograms (CVs) on carbon screen printed electrodes obtained using lysed blood sample and alkaline hematin respectively from the same blood sample (*see Paragraph-1 in* Supporting Information *for experimental details*). It is evident from the Fig. 1(c) that the lysed blood (Hb) does not show any electrochemical activity on a carbon printed electrode but alkaline hematin shows an irreversible CV as shown in Fig. 1(f). Although the reduction current of alkaline hematin appears to be a potential candidate for Hb sensing, it is not so in practice. The reduction current peak of alkaline hematin saturates at very low Hb concentration (micro-molar) range which is well below clinically relevant value (*see Paragraph-1 in* Supporting Information). The reason for this non-linear response is the formation of dimers of hematin in aqueous solution^32^,^33^. The dimerization of hematin hinders the diffusion controlled electrochemical reaction, which is highly desirable for biosensing.

The coordination chemistry of iron porphyrins has attracted considerable interest because of the widespread occurrence of the hemeproteins^34^. The origin of hemichromes was studied and classified in the late 60s. Hemichromes, with relatively high solubility in aqueous solutions, are low spin ferric compounds. They are formed when the 5^th^, 6^th^ coordination positions of iron covalently attach with a ligand. Reversible hemichrome can be formed with the coordination of internal ligands such as distal histidine with 6^th^ position of metal iron. Reversible hemichrome can convert back to the normal Hb biomolecule. The other types of hemichromes, called irreversible hemichromes, cannot be converted back to normal Hb. Irreversible hemichrome is characterized by the nitrogenous linkage at the 5^th^ and 6^th^ position of iron(III). Hemichromes have been shown to possess good diffusion controlled electrochemical activity without any complicated surface modifications^35^. This unique property of hemichrome is used here to develop Hb biosensor. We propose to convert Hb, into hemichrome by coordinating with nitrogenous ligands such as pyridine and imidazole. Then we obtain a well behaved reversible CV as opposed to the irreversible CV of alkaline hematin [Fig. 1(i,n)]. Further, nitrogenous ligands enable unhindered electron transfer with electrodes, as will be illustrated later.

### Pyridine liquid sensing chemistry based Hb detection

**Figure 2:**
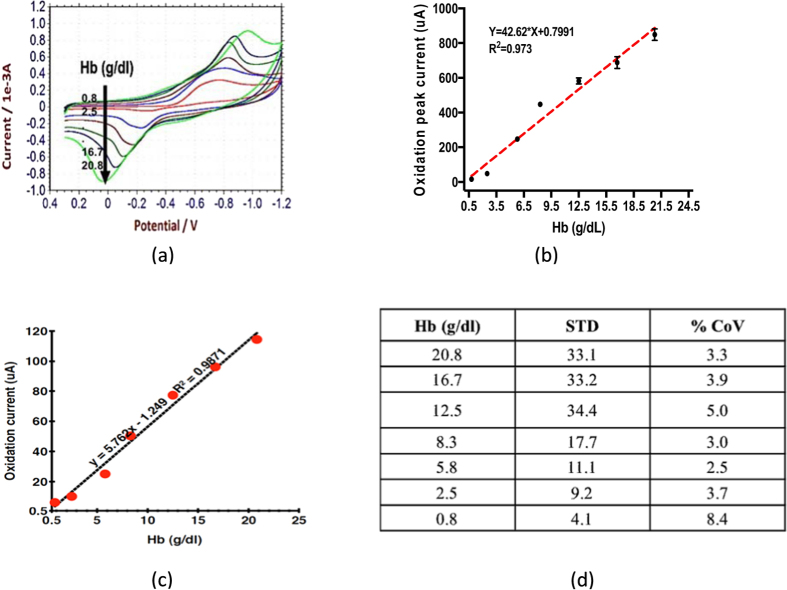
(**a**) CV of Pyridine hemichrome for different Hb conc. (**b**) Error bar plot of peak oxidation current Vs. Hb conc. (**c**) CA plot for Oxidation current Vs. Hb conc. (**d**) Standard deviations and Coefficients of Variation.

In pyridine based Hb detection, whole blood is lysed using DI water and is converted into alkaline hematin [Fig. 1(d)]. Alkaline hematin is then converted into pyridine hemichrome (pH = 12.9) after adding high concentration of pyridine in alkaline hematin solution [Fig. 1(g,i)]. The addition of DI water and pyridine solution dilutes the Hb concentration in original blood sample. This dilution is compensated after adding an extra Sigma Bovine hemin in the final solution, so that the final Hb concentration of the solution is 25 g/dL (*see Paragraph-2 in* Supporting Information *for experimental details*). 300 uL drop of different concentrations of Hb is applied at the carbon printed electrode and CVs are recorded for a wide range of Hb concentrations varying from 0.5 g/dl to 20.8 g/dl. Each concentration of Hb is tested three times on three different electrodes to analyze the repeatability of the assay. As seen in Fig. 2(a,b), the peak oxidation current is linearly proportional to the Hb concentration with R^2^ = 0.97. Instead of peak oxidation current, if we monitor the oxidation current at a fixed oxidation voltage of −0.06 V, the repeatability and linearity is equally good with R^2^ = 0.96 (*see* Fig. S11 *in* Supporting Information). Typically, Chronoamperometry (CA) is a preferred technique in POC devices. Hence we analyze the efficacy of CA in Hb sensing. As shown in Fig. 2(c), we get an excellent response with R^2^ = 0.98. (*see* Fig. S12 *in* Supporting Information *for CA graphs*). The formation of pyridine hemichrome, which enables this sensing scheme, is verified using the UV-VIS spectroscopy [*see Paragraph-3 in* Supporting Information *for details*]. In pyridine hemichrome experiment, chemicals such as NaOH and pyridine are used to convert Hb from lysed blood into pyridine hemichrome. In addition blood plasma may also contain many redox active molecules. Hence it is necessary to evaluate possible interference. The absence of redox peaks in CVs obtained in pure plasma solution, pyridine, NaOH confirmed the absence of any interference from the constituents of plasma, and other chemicals. The synthetic pyridine hemichrome samples prepared after adding pyridine into alkaline solution of Sigma Bovine hemin, produced redox peaks identical to human blood samples. This further confirmed that the proposed hemichrome conversion of Hb is solely responsible for the fully reversible redox activity [*see Paragraph-3 and* Fig. S3 *in* Supporting Information *for details*]. Figure 2(d) demonstrates that the standard deviation and coefficient of variations are well within the requirements for POC Hb biosensor. Thus the pyridine receptor chemistry is highly accurate and cost effective method for Hb sensing. But it would need some sample preparation protocol, due to its liquid nature.

### Imidazole dry sensing chemistry based Hb detection

For a robust POC biosensor, it is desirable to have stable, dry sensing chemistry on disposable carbon printed electrode. In imidazole based dry chemistry, Sodium dodecyl sulphate (SDS) and imidazole are functionalized on a paper membrane. SDS lyses the red blood cells (RBC) and also converts Hb into metHb by denaturation of protein (*see Paragraph-4 in* Supporting Information *for details*). Imidazole coordinates with iron (III) in porphyrin structure [Fig. 1(m)] and forms imidazole-metHb hemichrome complex, which gives the reversible CV [Fig. 1(n)]. A paper membrane, dispensed with SDS and imidazole based sensing chemistry, is laminated on the carbon printed electrodes. (*see Paragraph-5 in* Supporting Information *for details*).

**Figure 3:**
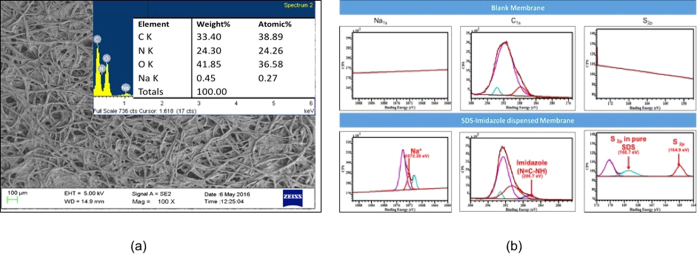
(**a**) SEM image of paper membrane dispensed with SDS-Imidazole chemistry (Inset *EDX spectra of the membrane*). (**b**) XPS wide band spectra of blank paper membrane and SDS-Imidazole paper membrane.

The paper membrane, functionalized with SDS-Imidazole sensing chemistry, is characterized by Scanning Electron Microscopy (SEM), Energy-dispersive X-ray spectroscopy (EDX) and X-ray Photoelectron Spectroscopy (XPS). Figure 3(a,b) show SEM, EDX and XPS spectra of the membrane after drying. The presence of sodium (Na) and nitrogen (N) in EDX spectra, clearly indicates signatures of SDS and imidazole. The XPS spectra shows the presence of sulphur (S), sodium (Na) and imidazole (*see Paragraph-5 in* Supporting Information *for details*).

**Figure 4:**
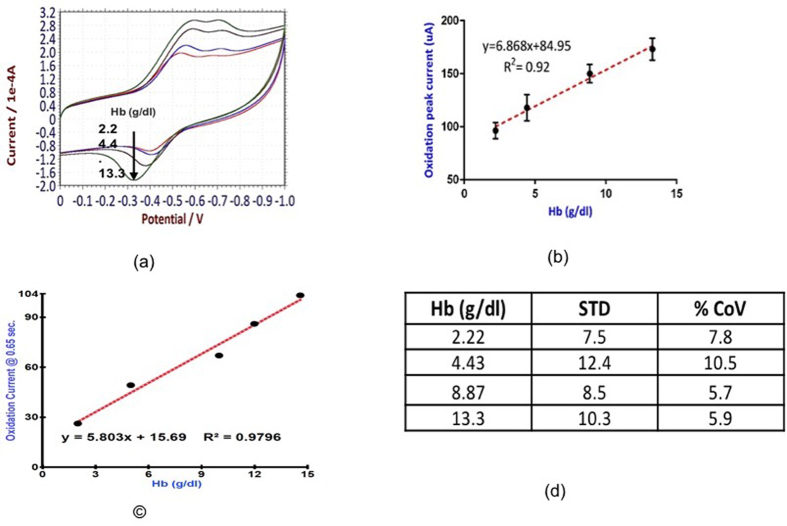
(**a**) CV of Imidazole hemichrome for different Hb conc. (**b**) Error bar plot of oxidation current Vs. Hb. (**c**) CA plot for Oxidation current Vs. Hb conc. (**d**) Standard deviation and Coefficients of Variation.

**Figure 5:**
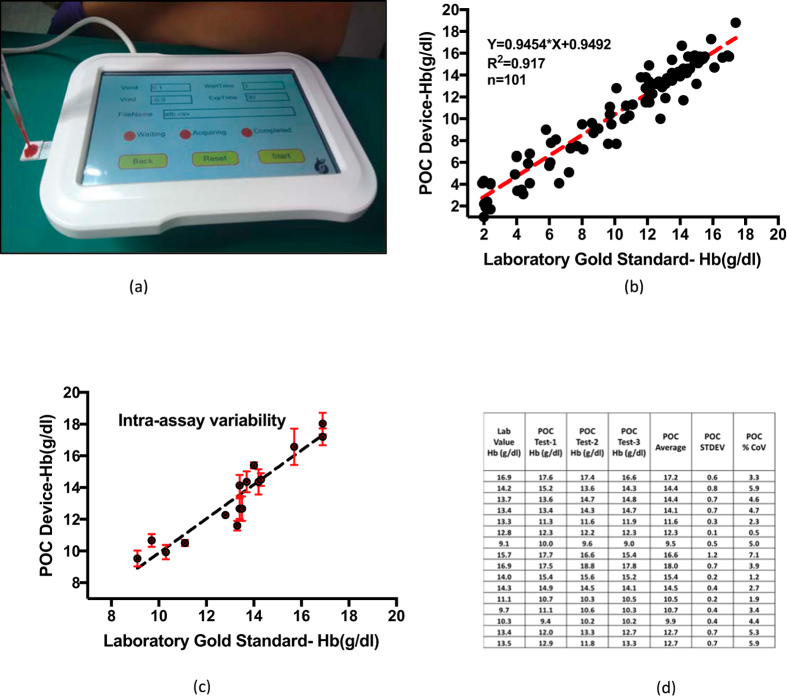
(**a**) Multi analyte electrochemical POC reader. (**b**) Correlation between Hb values of Laboratory Gold standard and SDS-Imidazole Dry chemistry using POC device. (**c**) Error bar plot for Intraassay variability. (**d**) Statistics for Intraassay variability.

300 uL drop of whole blood sample is applied on the membrane laminated printed carbon electrodes and CVs are recorded for four different Hb concentrations. Each concentration of Hb is repeated three times on three different test strips to estimate intra-assay variation. As seen in the Fig. 4(a,b), the oxidation current is proportional to Hb concentration in actual blood samples with R^2^ = 0.92. Figure 4(d) shows the standard deviation and coefficient of variation. The efficacy of SDS-imidazole dry chemistry has also been analyzed by CA (Fig. 4(c)). (*see* Fig. S13 *in* Supporting Information *for CA graphs*). From this data an algorithm is developed to map the oxidation current to the actual Hb concentration in blood sample. This algorithm is used to create a novel handheld POC Hb sensor (Fig. 5(a)). (*see Paragraph-6 in* Supporting Information *for details on handheld reader and disposable test strips*). We demonstrate excellent sensing capability of proposed POC sensor, through tests performed on 101 clinical samples (Fig. 5(b)). The POC tests on disposable test strips are performed using 70 uL of whole blood, with a testing time of about 30 seconds. The pathology lab tests are done using photometric method on Sysmex auto-analyzer using SLS-Hb complex. An excellent correlation with R^2^ = 0.917 is demonstrated between POC device and Sysmex results. The statistical bias was analyzed using Bland-Altman plot (*see* Fig. S14 *in* Supporting Information) and the POC device results are within 95% CI interval. (Figure 5(c,d) show the intra assay variability plot and statistics. For this analysis, each blood sample is tested using 3 different test strips. Further the intra-assay variability analysis indicates that the coefficient of variation is in the range of 0.5% to 7.1% In summary, this study represents a major step forward in realizing robust and scalable, direct electron transfer POC Hb sensor utilizing aza-heterocyclic receptors.

## Methods

### Reagents and chemicals

Sodium dodecyl sulfate (SDS) and bovine hemin (>90%) were procured from Sigma-Aldrich. Imidazole, Pyridine and Sodium Hydroxide were procured from Merck. Saline solution was purchased from Baxter (India) Pvt. Limited. All the above chemicals were commercially available and used as received without further purification.

### Apparatus and Measurements

Electrochemical experiments were performed on CHI Electrochemical Workstation 660E. SEM images and EDS spectra were captured on Carl Zeiss Ultra 55 FESEM. XPS measurements were done on AXIS ULTRA X-ray Photoelectron Spectroscopy UV-Spectroscopic measurements were performed on Shimadzu UV-Vis-IR Spectrophotometer. Carbon screen printed electrodes were used from PINE Instruments USA and GSI Technologies, USA with carbon material as working and counter and Ag/AgCl (PINE) or carbon (GSI) as reference.

### Pyridine hemichrome based sensing

In pyridine based Hb detection, 1.5 ml of whole blood (pH = 7.2) is lysed using 4 ml of cold DI water and is converted into alkaline hematin after adding 1.5 ml of 1 N NaOH. The pH of alkaline hematin solution is 12.6. Alkaline hematin is then converted into pyridine hemichrome (pH = 12.9) after adding 1.5 ml (12 M) of pyridine solution in alkaline hematin solution. The addition of DI water and pyridine solution dilutes the Hb concentration in original blood sample. This dilution is compensated after adding an extra 77 mg of Sigma Bovine hemin in the final solution, so that the final Hb concentration of the solution is 25 g/dL

### Imidazole hemichrome based sensing

The stock solution of 69 mM SDS and 7.4 M imidazole is prepared in DI water and the final pH of the solution was 10.9. Blank paper membrane is laminated on the carbon printed electrodes. A micro droplet of 100 uL of SDS-imidazole based sensing chemistry is dispersed on paper membrane and dried at 30 °C for 90 minutes with a forced airflow at a velocity of 1.5 m/sec.

## Additional Information

**How to cite this article**: Kumar, V. *et al*. Aza-heterocyclic Receptors for Direct Electron Transfer Hemoglobin Biosensor. *Sci. Rep.* **7**, 42031; doi: 10.1038/srep42031 (2017).

**Publisher's note:** Springer Nature remains neutral with regard to jurisdictional claims in published maps and institutional affiliations.

## Supplementary Material

Supplementary Information
