# Supplementary material for: Aza-heterocyclic Receptors for Direct Electron Transfer Hemoglobin Biosensor
Source: Sci Rep. 2017 Feb 7;7:42031. doi: 10.1038/srep42031 (PMC5294641; doi:10.1038/srep42031)
Supplement: Supplementary Information [file srep42031-s1.pdf]

Aza-heterocyclic Receptors for Direct Electron Transfer Hemoglobin Biosensor

Vinay Kumar[1,2], Nikhila Kashyap D.M.[2], Suraj Hebbar[2], Swetha R.[3], Sujay Prasad[3], Kamala T.[4], S.S Srikanta[4], P.R. Krishnaswamy[1], and Navakanta Bhat[1]

Centre for Nano Science and Engineering, Indian Institute of Science, Bangalore[1]

PathShodh Healthcare Pvt Ltd, Bangalore[2]

Anand Diagnostics Laboratory, Bangalore[3]

Samatvam Diabetes Endocrinology Centre, Bangalore[4]

Reagents and chemicals: Sodium dodecyl sulfate (SDS) and bovine hemin (>90%) were procured from Sigma-Aldrich. Imidazole, Pyridine and Sodium Hydroxide were procured from Merck. Saline solution was purchased from Baxter (India) Pvt. Limited. All the above chemicals were commercially available and used as received without further purification.

Apparatus and Measurements:

- Electrochemical experiments were performed on **CHI Electrochemical Workstation 660E.**
- SEM images and EDS spectra were captured on **Carl Zeiss Ultra 55 FESEM.**
- XPS measurements were done on **AXIS ULTRA** X-ray Photoelectron Spectroscopy
- UV-Spectroscopic measurements were performed on **Shimadzu** UV-Vis-IR Spectrophotometer.
- Carbon screen printed electrodes were used from **PINE Instruments USA and GSI Technologies, USA** with carbon material as working and counter and Ag/AgCl (PINE) or carbon (GSI) as reference.

In-Vitro Experiments: All the experiments and procedures involving blood and clinical samples were conducted with the approval of Ethical committee.

1. **Paragraph-1 (Alkaline hematin)**

Lysing of EDTA mixed whole blood (pH 7.21) was carried out by adding 4 ml of cold DI water into 1.5 ml of whole blood with Hb concentration of 14.6 g/dl. The pH of the lysed blood solution was 7.39. Hemoglobin in this 5.5 ml lysed sample was converted into 7 ml alkaline hematin after adding the 1.5 ml 1N NaOH with final solution pH of 12.63. The CV plot has been recorded by using 300 ul drop of alkaline hematin solution on carbon printed electrode.

Figure S1, shows the CVs of alkaline hematin. Initially the peak reduction current of alkaline hematin increases but it saturates even at very lower concentrations of hemoglobin.

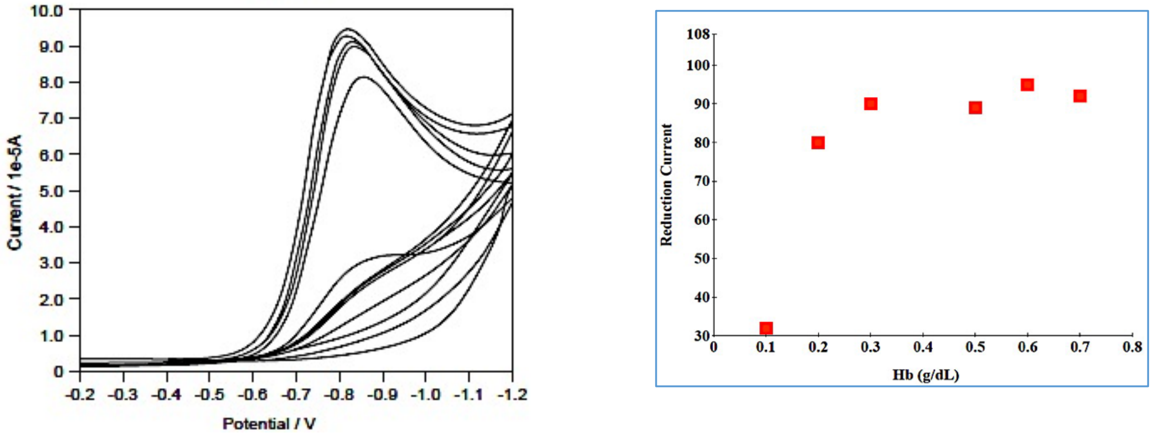

Figure S1 (a) CV of hemoglobin after it is converted into alkaline hematin (b). Peak reduction current Vs. Hb concentration

2. Paragraph-2 (Pyridine hemichrome)

7 ml alkaline hematin (initial Hb in whole blood = 14.6 g/dl) solution (as described in ***Paragraph-1***) was converted into pyridine hemichrome by adding 1.5 ml (12 M) pyridine solution. The final volume and pH of pyridine hemichrome solution was 8.5 ml and 12.9 respectively. The final concentration of Hb in 8.5 ml of pyridine hemichrome, after dilution, was 2.5 g/dl. To compensate this dilution of Hb, 77 mg of standard Sigma bovine hemin was added to the 8.5 ml sample solution which corresponded to total Hb concentration of 25 g/dl in the final solution. This solution was diluted with saline accordingly to get a wide range of Hb concentration from 2.5 g/dl to 20.8 g/dl for electrochemical measurements. Cyclic voltammetry (CV) parameters were chosen as follows: scan rate 0.6 V/s, potential window 0.3 V to -1.2 V; Chronoamperometry (CA) parameters were chosen as follows: oxidation voltage was -0.06V and reduction voltage was -0.88 V, pulse width of 5 sec.

3. Paragraph-3

The UV spectroscopic analysis of formation of pyridine hemichrome from whole blood was carried:

A. UV absorption spectrum of lysed blood (4 ml of cold DI water into 1.5 ml of whole blood).

B. Alkaline hematin was obtained from A by adding 1.5 ml of 1N NaOH solution to lysed blood in A. Then pyridine hemichrome was obtained by adding (1.5 ml (12 M) of pyridine solution.

C. UV absorption spectrum of pyridine hemichrome converted from B (1.5 ml (12 M) of pyridine solution added in the solution of step B).

10 ul drop of each of above samples were mixed in 3.2 ml of saline in cuvette to obtain the UV spectra of lysed blood (hemoglobin) and pyridine hemichrome. As shown in Figure S2, absorbance peaks of hemoglobin are located at 416 nm, 540 nm and 570 nm. The decreasing absorbance at 416 nm and evident disappearance of two smaller peaks at 540 nm and 570 nm in the plots suggest the conversion of hemoglobin to pyridine hemichrome. [1-2]

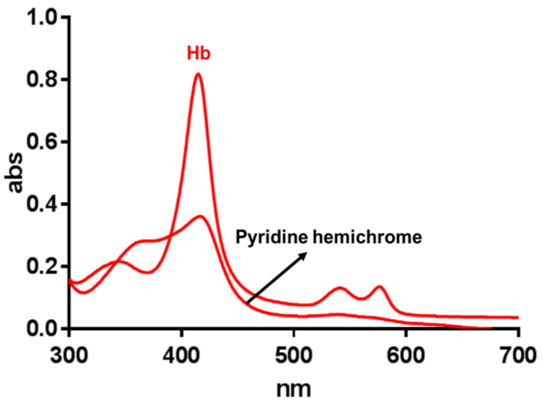

Figure S2: UV spectra of lysed blood (hemoglobin) and pyridine hemichrome

In pyridine hemichrome experiment, hemoglobin from lysed blood is converted into pyridine hemichrome. To make sure that the redox peaks in CVs is essentially due to Hb converted into hemichrome, and there is no interference from either the chemicals used to obtain pyridine hemichrome or other constituents of the plasma, CVs are performed on carbon electrodes. As shown in Fig. S3, the CVs of blood plasma, NaOH and Pyridine recorded on carbon SPEs using 300 uL of sample volume, do not show any redox peaks similar to the pyridine hemichromes. Further, the synthetic pyridine hemichrome samples have been prepared after adding the pyridine into alkaline solution of Sigma Bovine hemin. The pyridine hemichrome prepared from Bovine hemin also shows the redox activity which is exactly identical to CVs obtained from real blood samples.

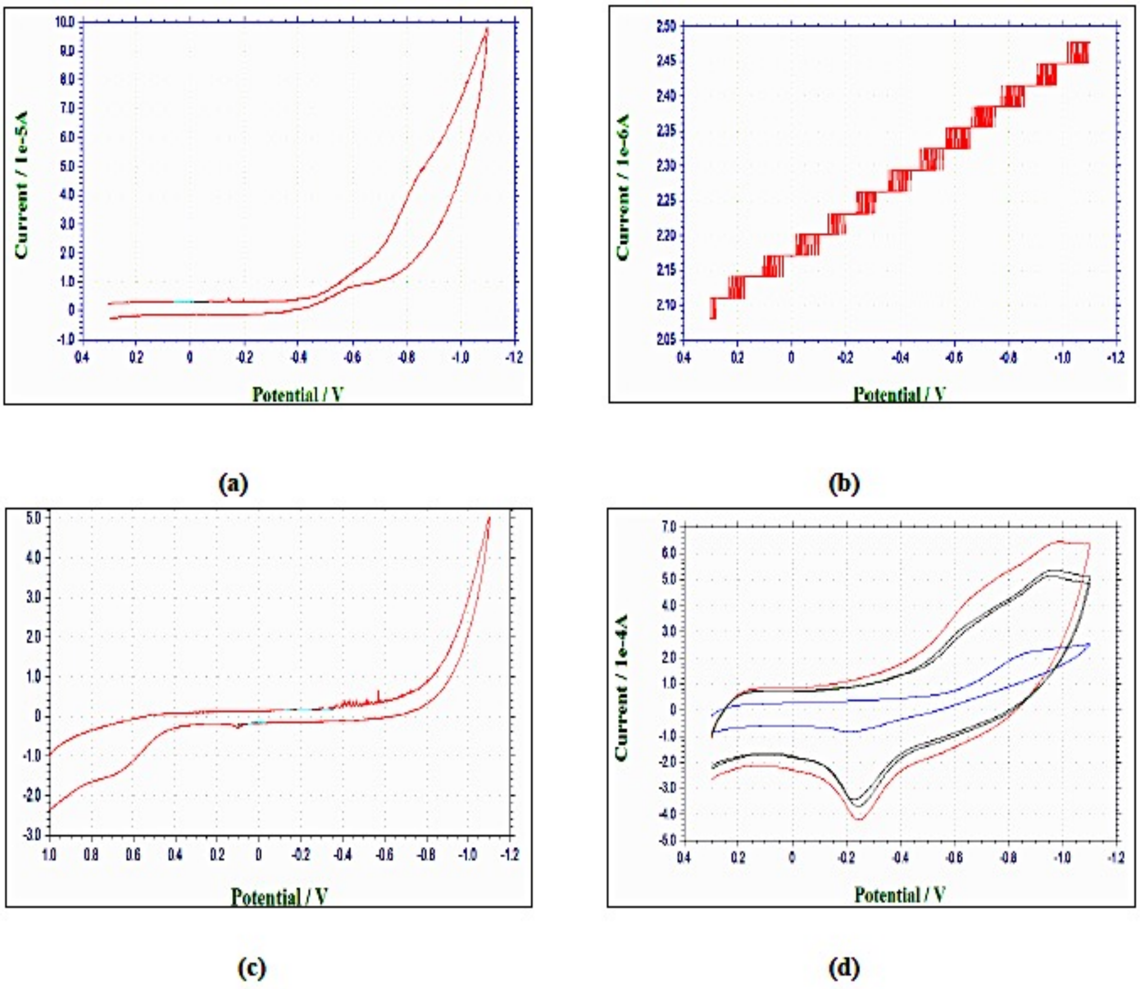

Figure S3 (a) CV of 1% NaOH on carbon SPE (b). CV of pyridine on carbon SPE (c). CV of blood plasma on carbon SPE (d). CV of synthetic pyridine hemichrome prepared from Sigma Bovine hemin

4. Paragraph-4 (Use of SDS)

The UV spectroscopic analysis of SDS based lysing of whole blood was carried. 69 mM SDS solution was prepared in saline solution. 200 ul of this solution was added in 200 ul of whole blood. 10 ul drop of this sample was mixed in 3.2 ml of saline in cuvette to obtain the UV spectra of lysed blood (hemoglobin). UV spectrum of SDS lysed blood shows similar UV-profile (Figure S4 (a)) as that of water lysed blood (Figure S4 (b)) with absorbance peaks of hemoglobin located at 416 nm, 540 nm and 570 nm in both the UV-Spectra.

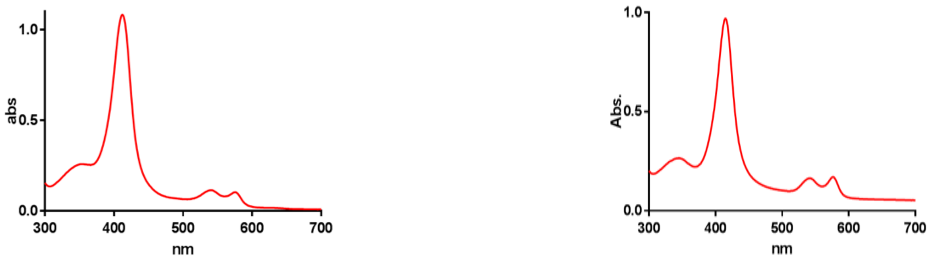

Figure S4 UV-spectra of (a). Whole blood lysed by SDS and (b). Whole blood lysed by cold DI water

In imidazole based chemistry, SDS was used for lysing the RBCs as well as to oxidize the hemoglobin into methemoglobin form. SDS, due to its solid form, can easily be dispersed and dried onto the membrane. The CV of hemoglobin lysed with SDS in Figure S5 (a) shows the similar profile as that of alkaline hematin in Figure S5 (b). SDS denatured the structure of hemoglobin and facilitates the easy access of the iron (III) to the carbon screen printed electrode with an irreversible reduction peak. This observation suggests that SDS not only lyses RBCs, but also denatures and oxidizes the hemoglobin into methemoglobin.

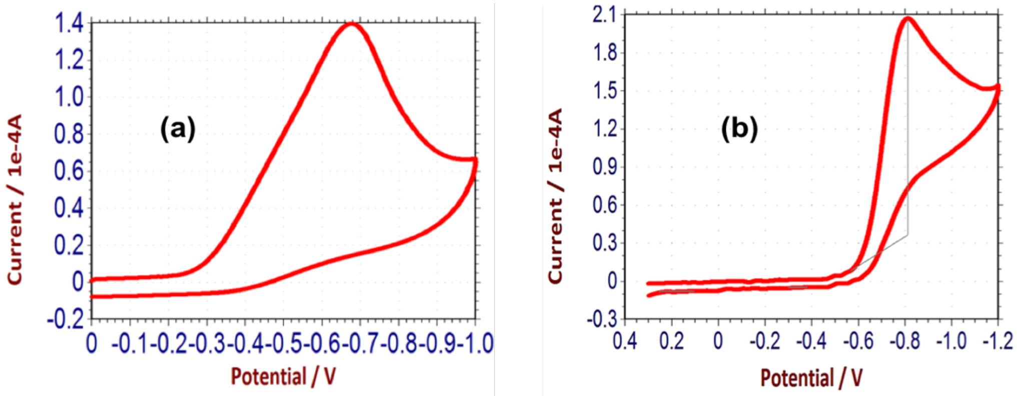

Figure S5 CV of (a) Whole blood lysed by SDS (b) Whole blood converted into alkaline hematin

5. Paragraph-5 (SEM, EDS and XPS characterization of membrane)

Filter paper membranes are used to hold the dry chemistry in SDS-Imidazole based detection, as shown in Figure S6. FIG S7 (a) and FIG S7 (b) show the SEM images and EDS spectra of blank undispersed membrane. The average pore size of the undispersed membrane is in the order of 10-20 micron with no signature of Imidazole (% N content is zero), SDS (% Na content is zero). On the other side, after the dispersion and drying the SDS-Imidazole chemistry EDS shows the nitrogen and sodium contents which are the signature of the presence of Imidazole and SDS, as shown in Figure 4.

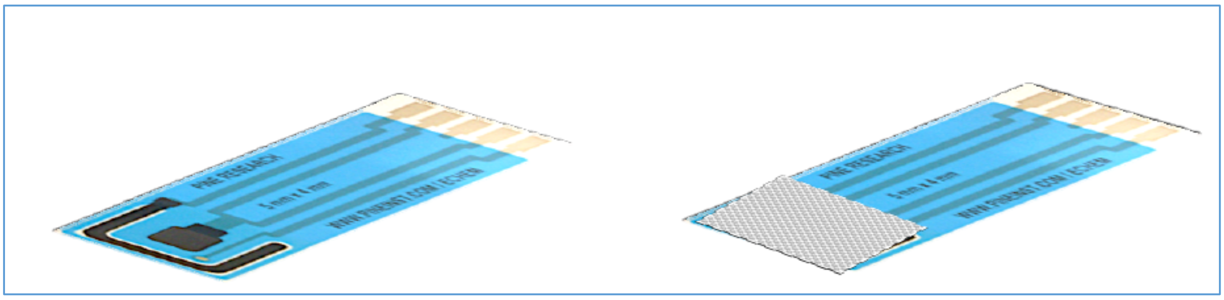

Figure S6 (a) blank carbon screen printed electrode(SPE) (b). Carbon SPE with filter paper membrane dispersed with sensing chemistry

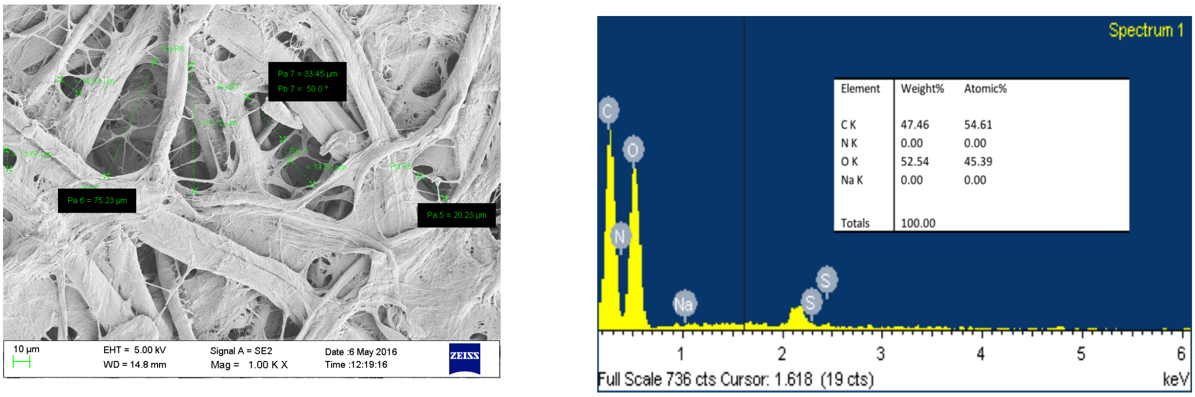

Figure S7 (a) SEM image of blank cotton membrane (b) EDS spectrum of blank cotton membrane

Figure S8 shows the wide band spectra of filter paper membrane dispensed with SDS and Imidazole chemistry.

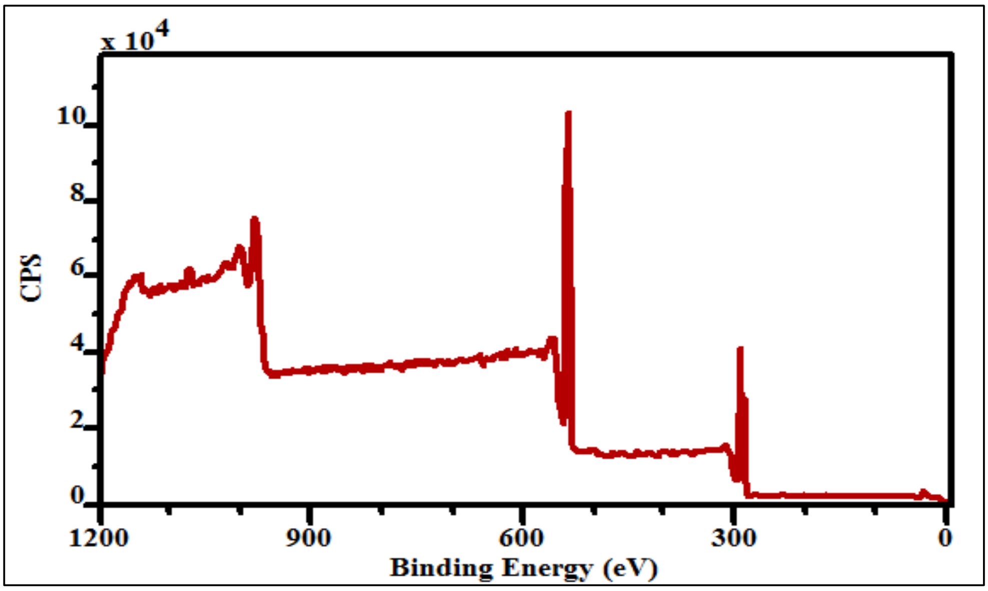

Figure S8 Wide band XPS spectra of filter paper membrane functionalized with SDS-Imidazole chemistry

Figure S9 shows the XPS spectra of sulfur (S 2p), sodium (Na 1s) and carbon (C 1s) for blank paper membrane and for SDS-Imidazole dispensed and paper membrane. Sodium dodecyl sulphate (SDS) contains sulphur. Sulfur shows the XPS peak at binding energy (BE) 164.9 eV and 168.7 eV while there is no S 2p spectra on blank paper membrane. The higher binding energy peaks in the spectra corresponding to the sulfur bonding with oxygen and sodium in the structure of SDS. [7] XPS spectra of sodium (Na) which is also present in the structure of SDS shows its XPS peak at BE 1072.6 eV which corresponds to Na^{+}. After comparing the C1s spectra in blank membrane and paper membrane dispensed with SDS-Imidazole a small peak at around 286.7 eV corresponding to the N=C-NH bonding in the imidazole structure is clearly seen on membrane dispersed with imidazole.

The other Carbon peaks at higher BE (such as 290-292 eV in C 1s), found in both membranes may be due to the carbonates (CO_3) which are generally used in paper manufacturing and present in the paper membrane. [3-7]

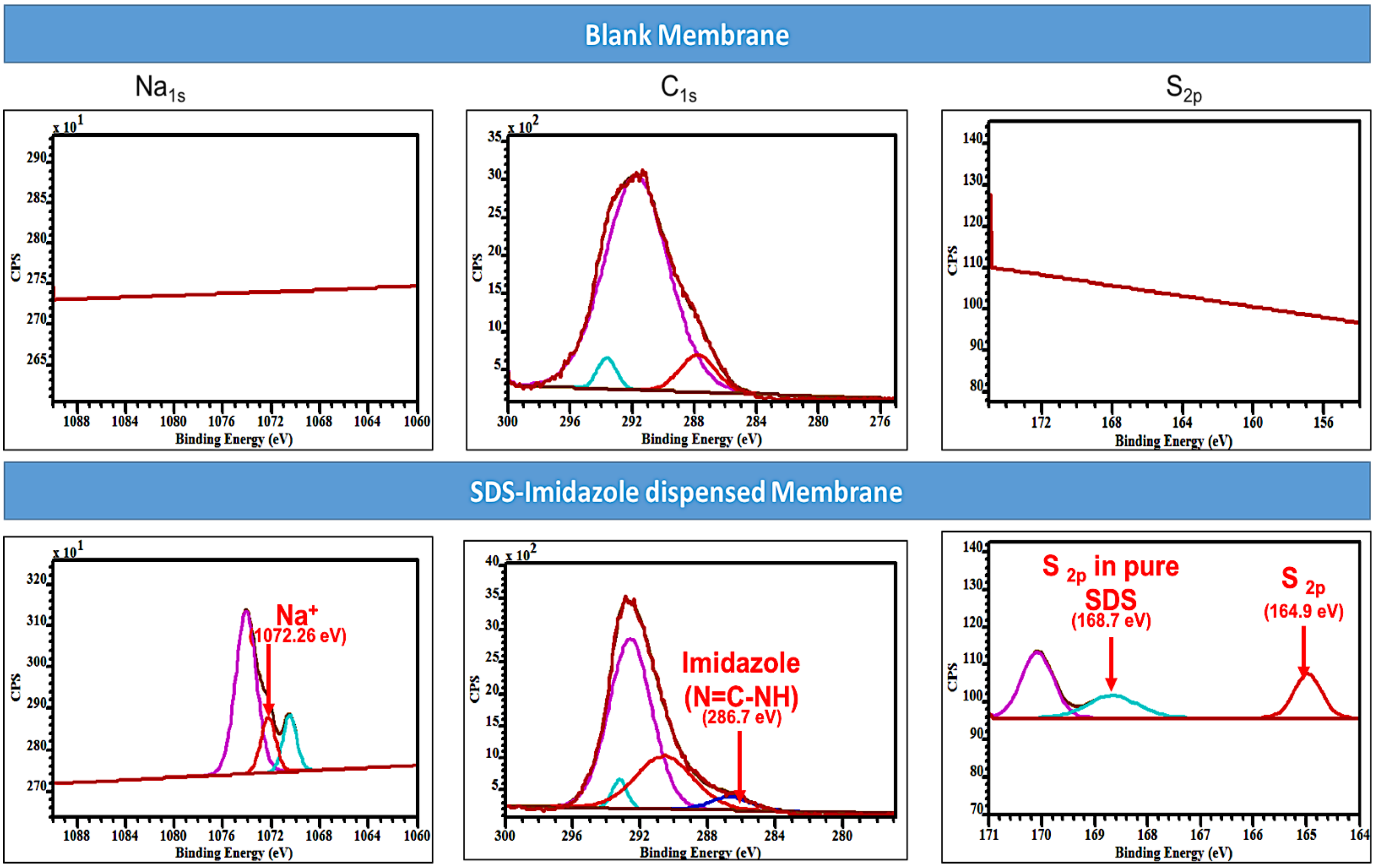

Figure S9 XPS spectra of: Na_{1p}, S_{2p}, C_{1s} in blank paper membrane and SDS-Imidazole dispensed membrane

6. Point-of-care (POC) hand held device

A POC device has been developed in-house and the aza-heterocyclic based sensing chemistry of SDS-imidazole has been verified this system using the GSI carbon screen printed electrodes. Figure S10 (a) shows the unpackaged device with PCB and other components. Figure S10 (b) shows the fully packaged device.

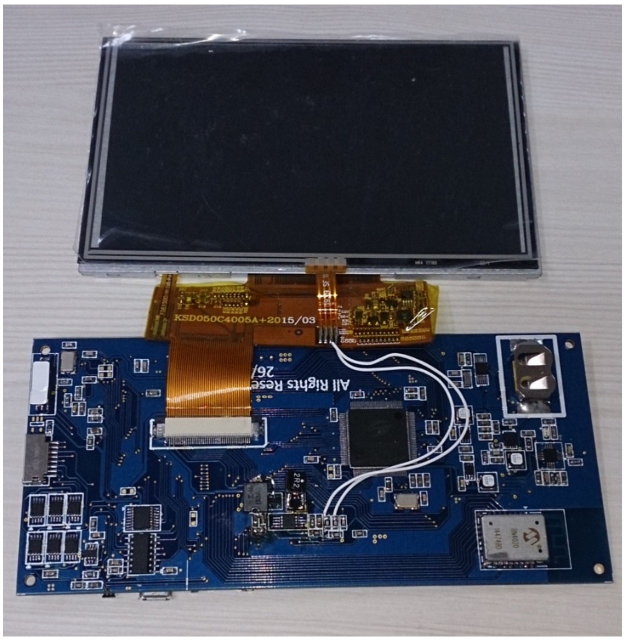

Figure S10 (a) Internal viewed of unpackaged in-housed developed POC device

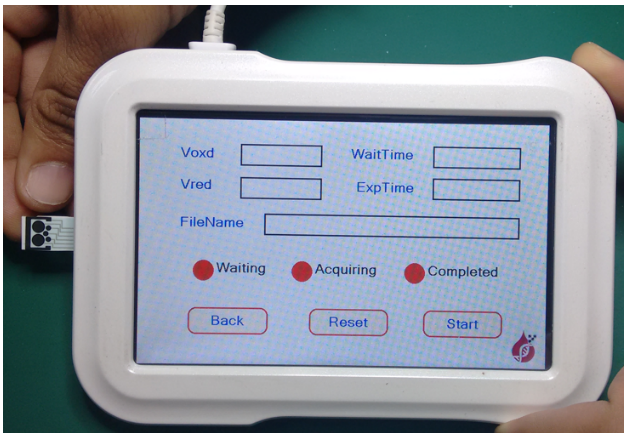

Figure S10 (b) Fully packaged POC device with test strip

7. Redox current versus Hb concentration at a fixed potential from CV

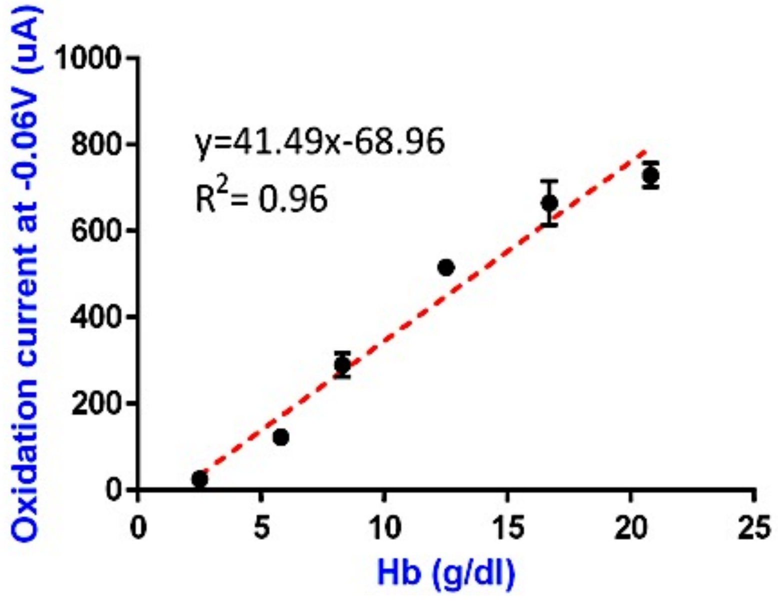

Figure S11 Error bar plot of oxidation current @ -0.06 V Vs. Hb concentration

8. Chronoamperometry (CA) plots

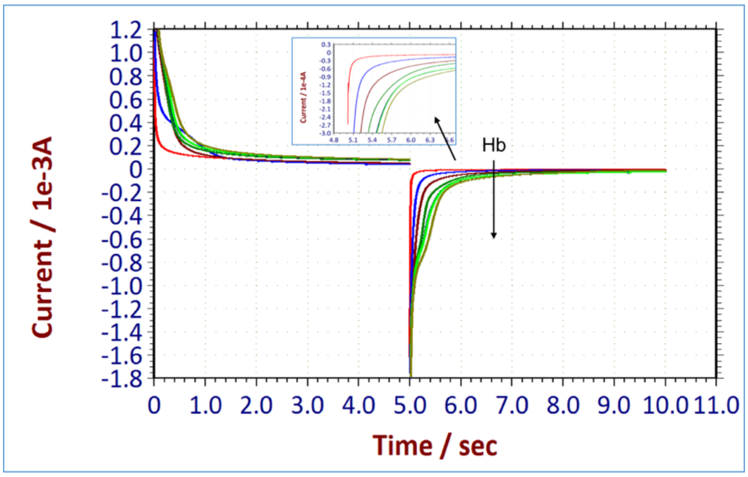

Figure S12 CA plots for Pyridine hemichrome

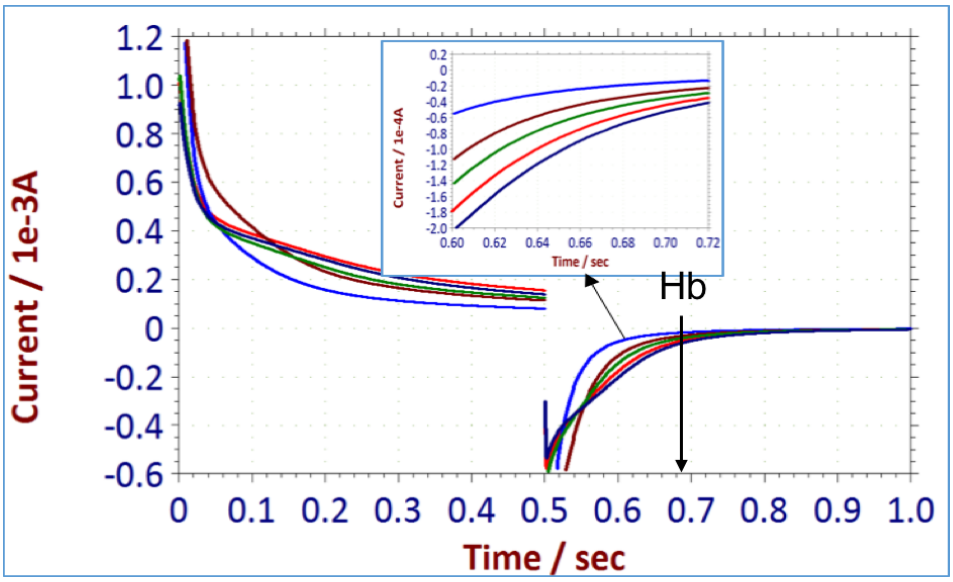

Figure S 13 CA plots for imidazole hemichrome

9. Bland-Altman plot

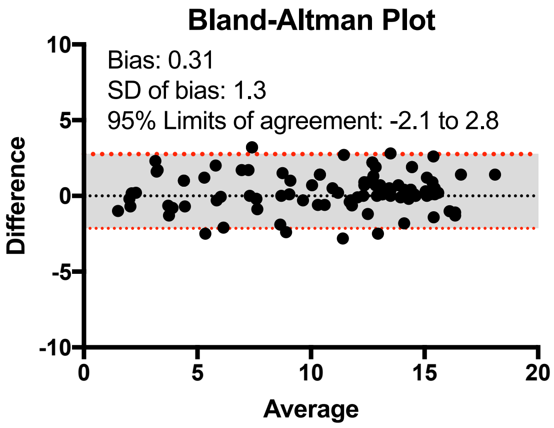

Figure S14 Bland-Altman plot

References

1. Edward A. Berry et. al.; Analytical Biochemistry, **1987**, 161, l-15.
2. Kanyile K. Ncokazi et. al; Analytical Biochemistry, **2005**, 338, 306–319.
3. John F. Moulder; Handbook of X-ray Photoelectron Spectroscopy: A Reference Book of Standard Spectra for Identification and Interpretation of XPS Data, **1992**, Perkin-Elmer Corporation.
4. Gi Xue, Quinpin Dai and Shuangen Jiang; Chemical Reactions of Imidazole with Metallic Silver Studied by the Use of SERS and XPS Techniques; J. Am. Chem. SOC., 1988, Vol. 110, No. 8.
5. Joanna S. Stevens et al; Quantitative analysis of complex amino acids and RGD peptides by X-ray photoelectron spectroscopy (XPS); Surface and Interface Analysis; 2-13, Volume 45, Issue 8, Pages 1238–1246.
6. Thermo Fisher Scientific Inc. (*http://xpssimplified.com/index.php*)
7. Johannes G.A. Terlingen, "Immobilization of surface active compounds on polymers supports using glow discharge processes 1. Sodium dodecyl sulphate on poly (propelene)", Journal of colloid and interface secience,1993, 155,55,(65).

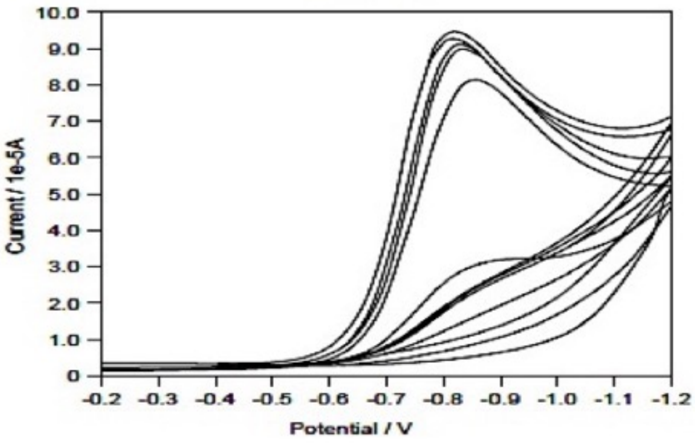
10.0
9.0
8.0
7.0
6.0
5.0
4.0
3.0
2.0
1.0
0
Current / 1e-5A
-0.2 -0.3 -0.4 -0.5 -0.6 -0.7 -0.8 -0.9 -1.0 -1.1 -1.2
Potential / V

(a)

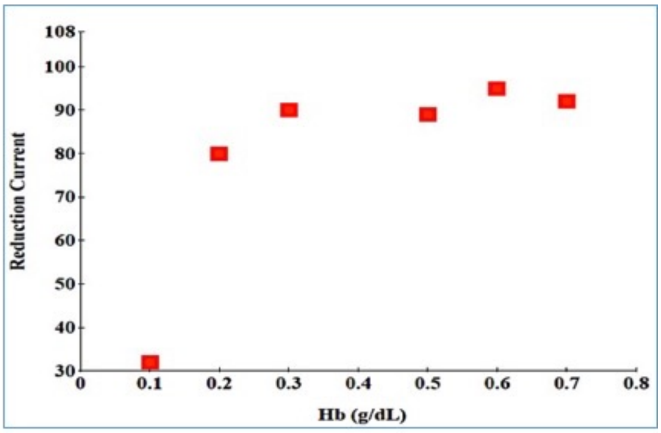
108
100
90
80
70
60
50
40
30
Reduction Current
0 0.1 0.2 0.3 0.4 0.5 0.6 0.7 0.8
Hb (g/dL)

(b)

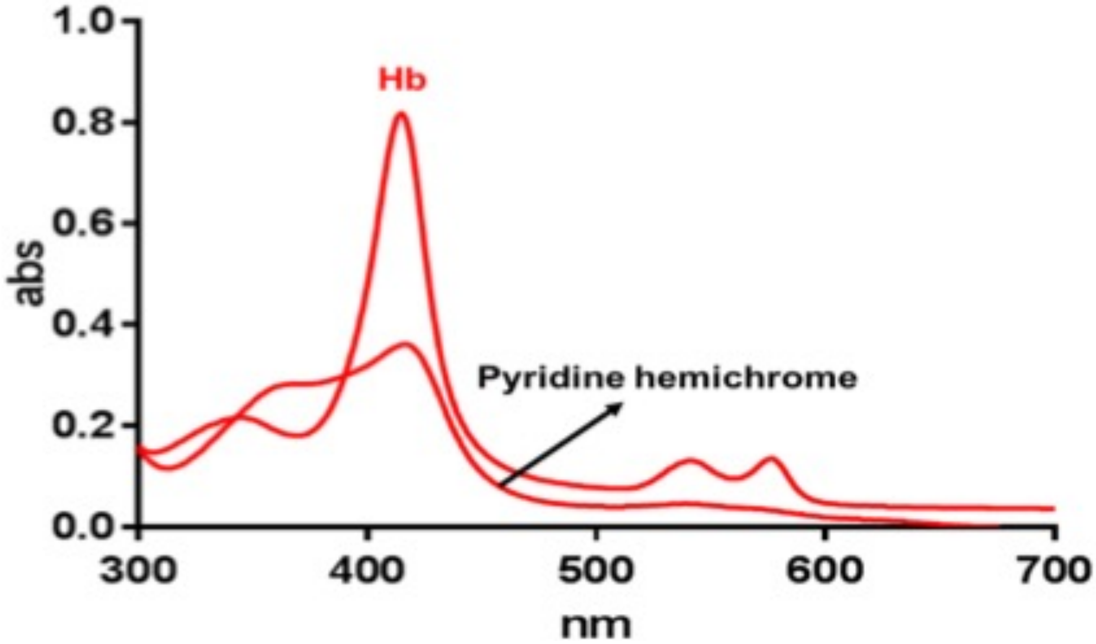

1.0
0.8
0.6
0.4
0.2
0.0
abs
Hb
Pyridine hemichrome
300
400
500
600
700
nm

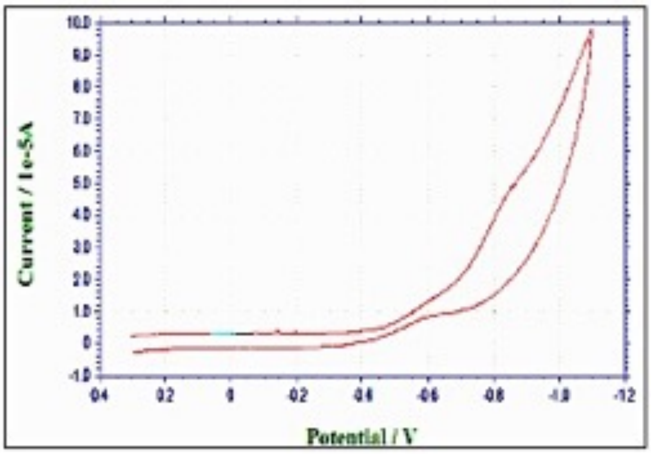
Current / 1e-5A
Potential / V

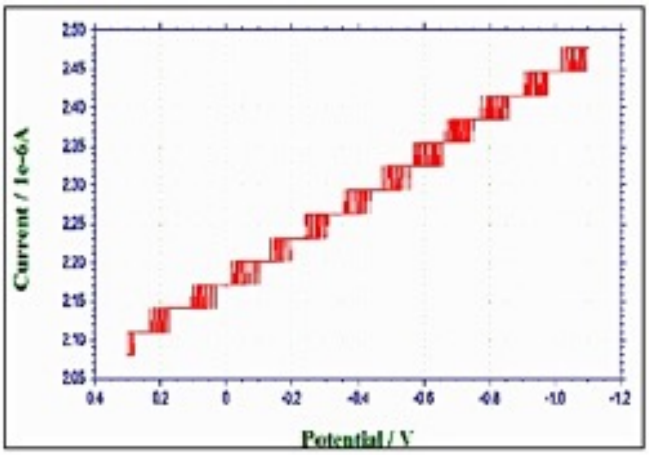
Current / 1e-6A
Potential / V

(a) **(b)**

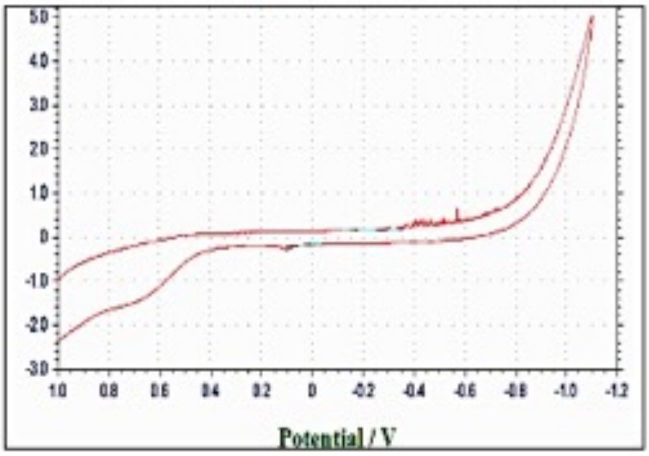
Potential / V

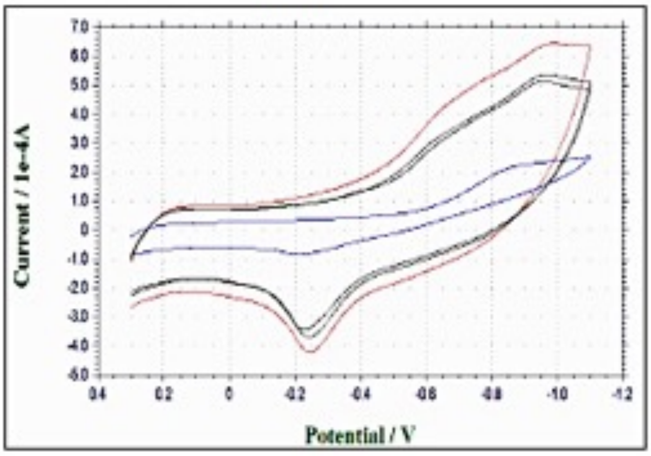
Current / 1e-4A
Potential / V

(c) **(d)**

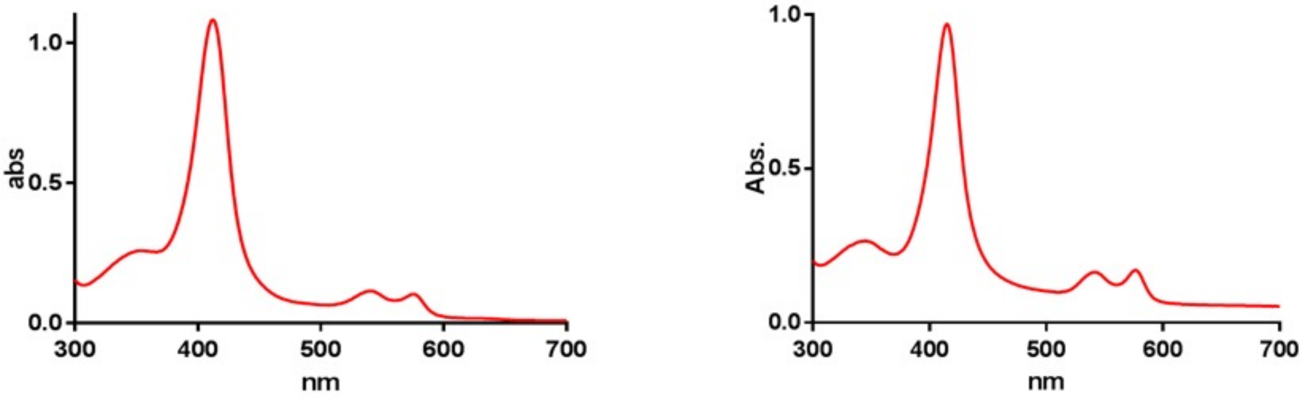

abs
1.0
0.5
0.0
300
400
500
600
700
nm
Abs.
1.0
0.5
0.0
300
400
500
600
700
nm

(a) (b)

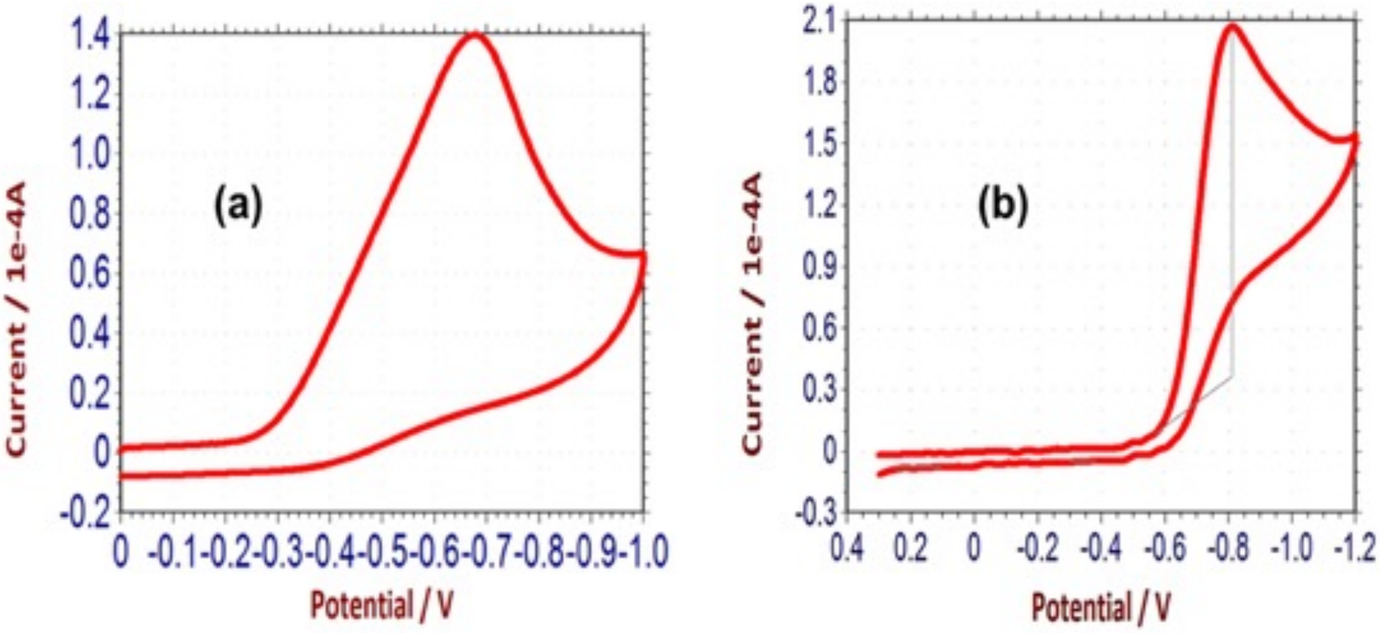

(a)
Current / 1e-4A
1.4
1.2
1.0
0.8
0.6
0.4
0.2
0
-0.2
0 -0.1 -0.2 -0.3 -0.4 -0.5 -0.6 -0.7 -0.8 -0.9 -1.0
Potential / V
(b)
Current / 1e-4A
2.1
1.8
1.5
1.2
0.9
0.6
0.3
0
-0.3
0.4 0.2 0 -0.2 -0.4 -0.6 -0.8 -1.0 -1.2
Potential / V

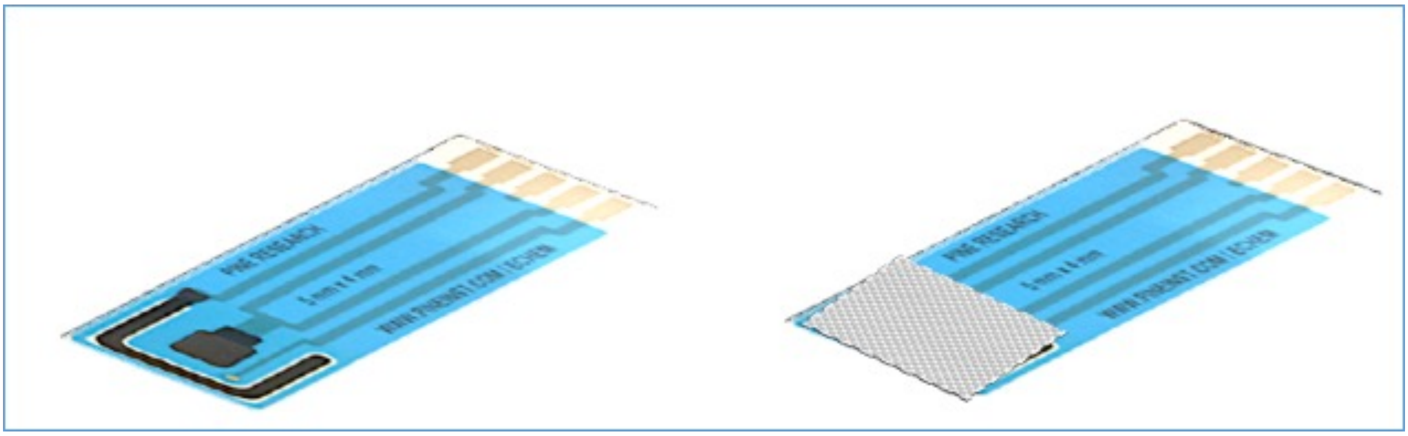
PINE RESEARCH

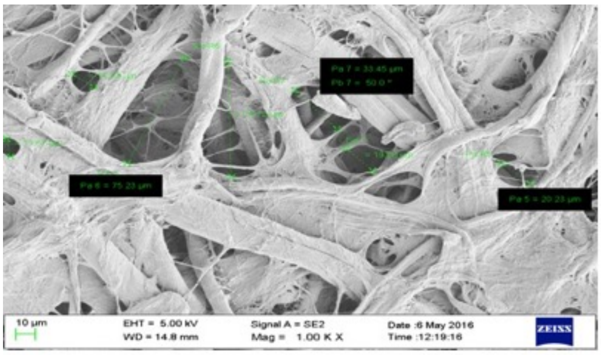

10 µm
EHT = 5.00 kV
WD = 14.8 mm
Signal A = SE2
Mag = 1.00 K X
Date :6 May 2016
Time :12:19:16
ZEISS

(a)

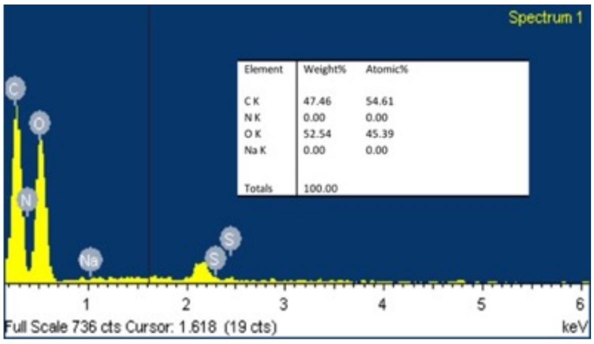

Spectrum 1
Element Weight% Atomic%
C K 47.46 54.61
N K 0.00 0.00
O K 52.54 45.39
Na K 0.00 0.00
Totals 100.00
C
O
N
Na
S
S
1 2 3 4 5 6
Full Scale 736 cts Cursor: 1.618 (19 cts)
keV

(b)

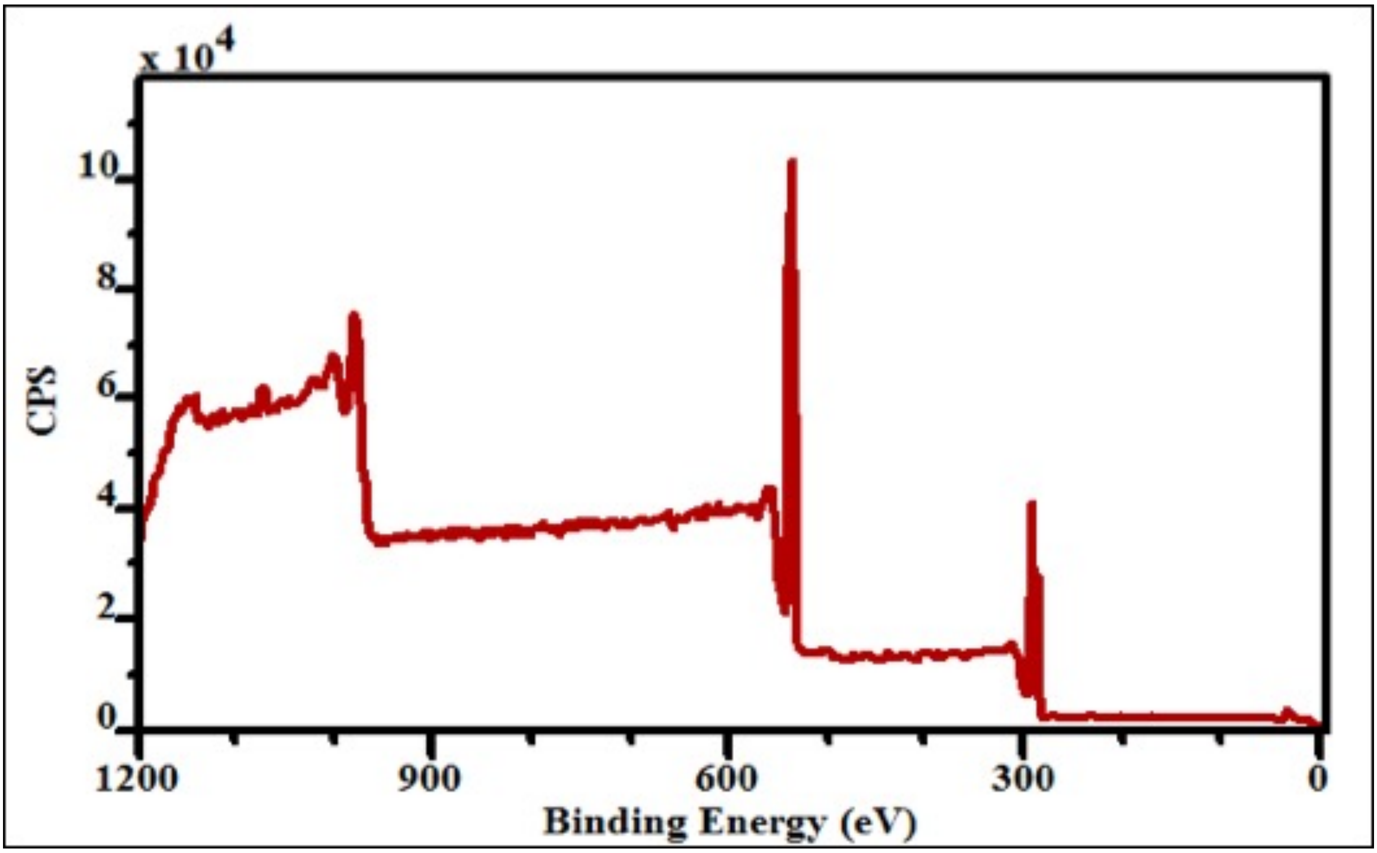
x 10^4
CPS
0
2
4
6
8
10
1200
900
600
300
0
Binding Energy (eV)

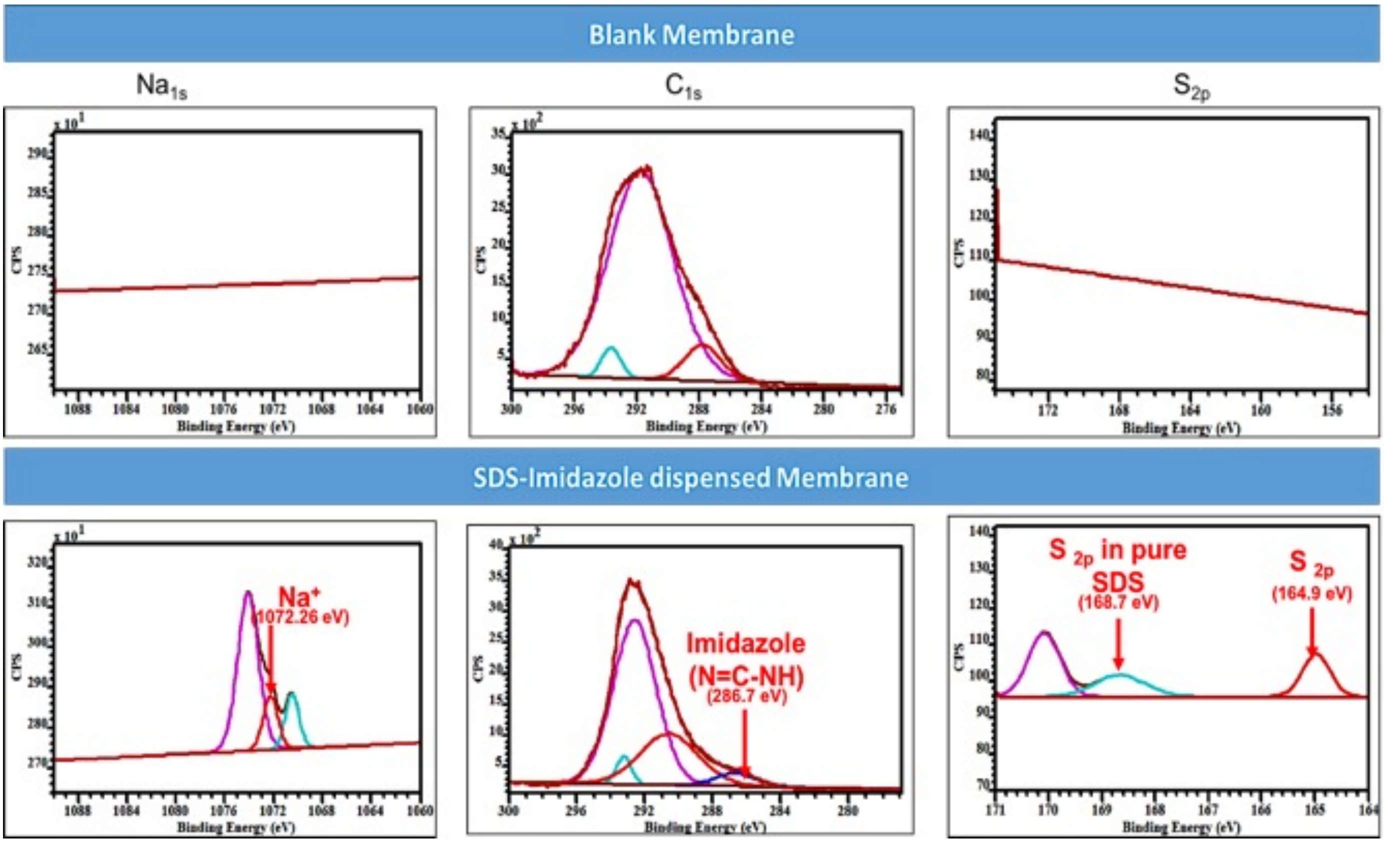

Blank Membrane
Na1s
C1s
S2p
Binding Energy (eV)
CPS
SDS-Imidazole dispensed Membrane
Na+
(1072.26 eV)
Imidazole
(N=C-NH)
(286.7 eV)
S 2p in pure SDS
(168.7 eV)
S 2p
(164.9 eV)

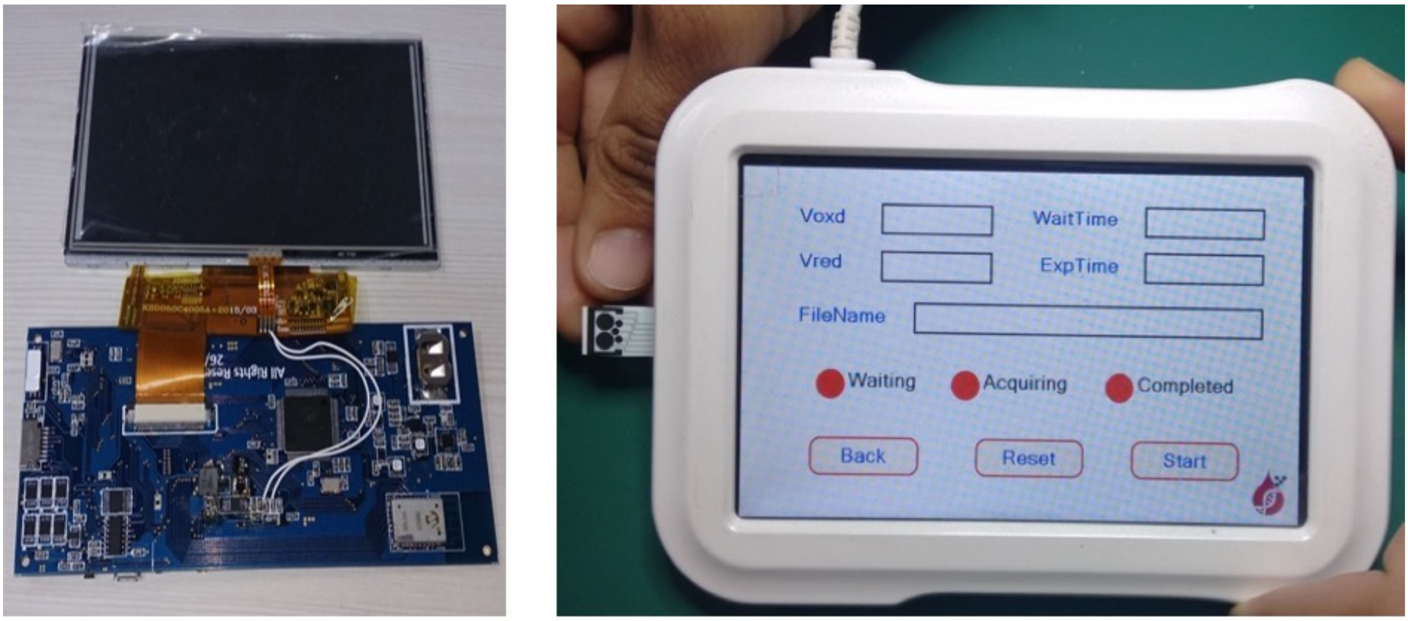
Voxd
WaitTime
Vred
ExpTime
FileName
Waiting
Acquiring
Completed
Back
Reset
Start

(a) (b)

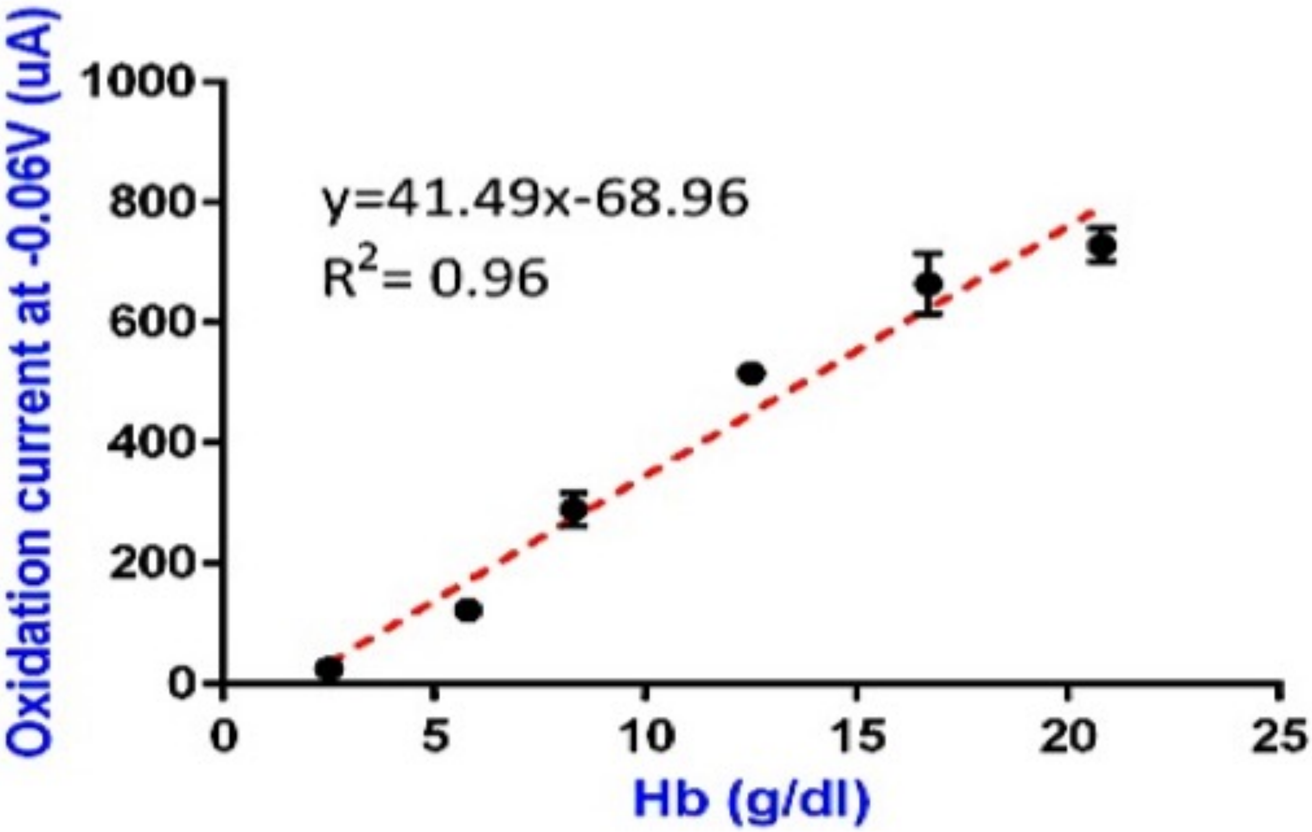

Oxidation current at -0.06V (uA)
1000
800
600
400
200
0
0
5
10
15
20
25
Hb (g/dl)
y=41.49x-68.96
R2= 0.96

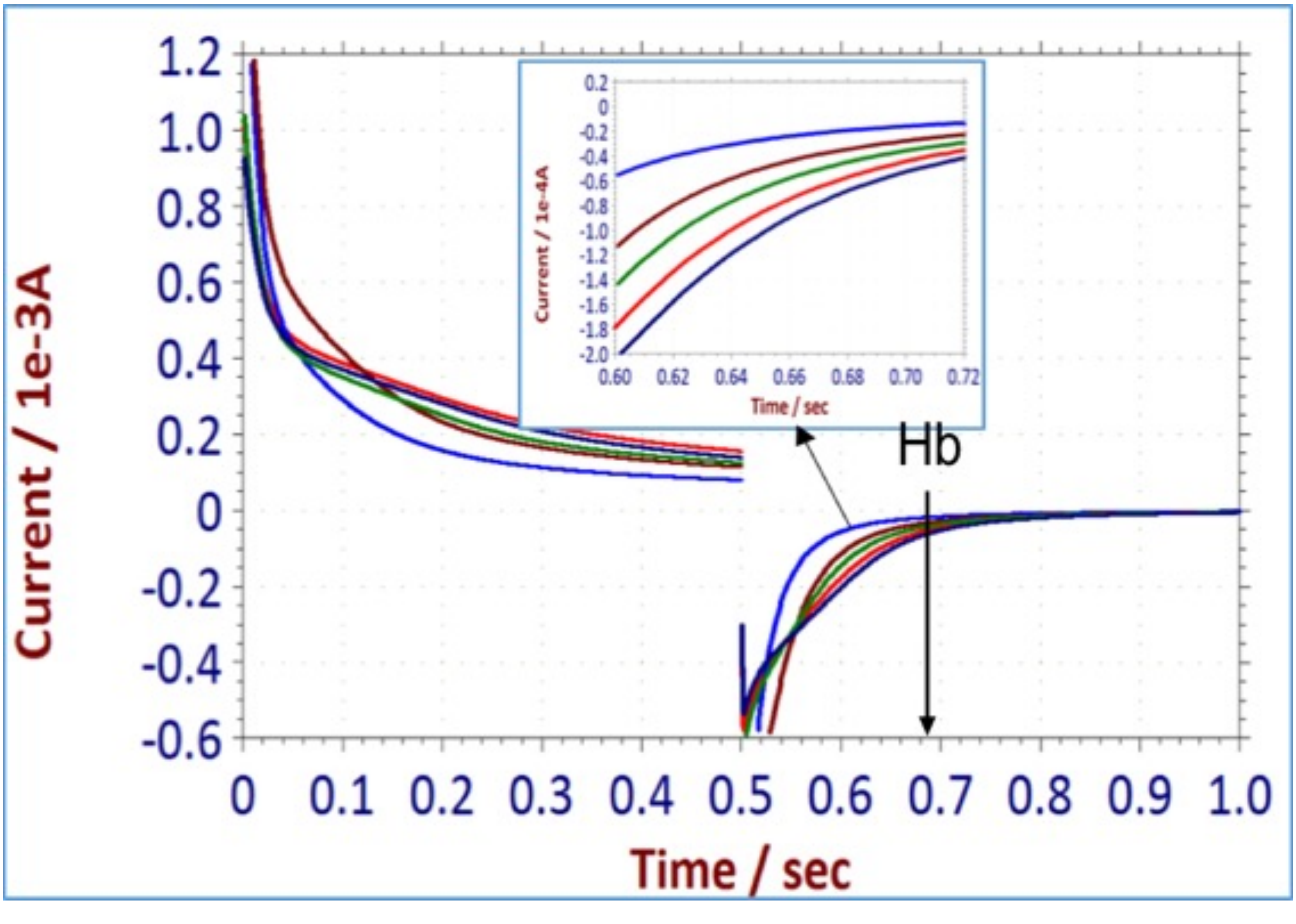

Current / 1e-3A
Time / sec
Hb
Current / 1e-4A
Time / sec

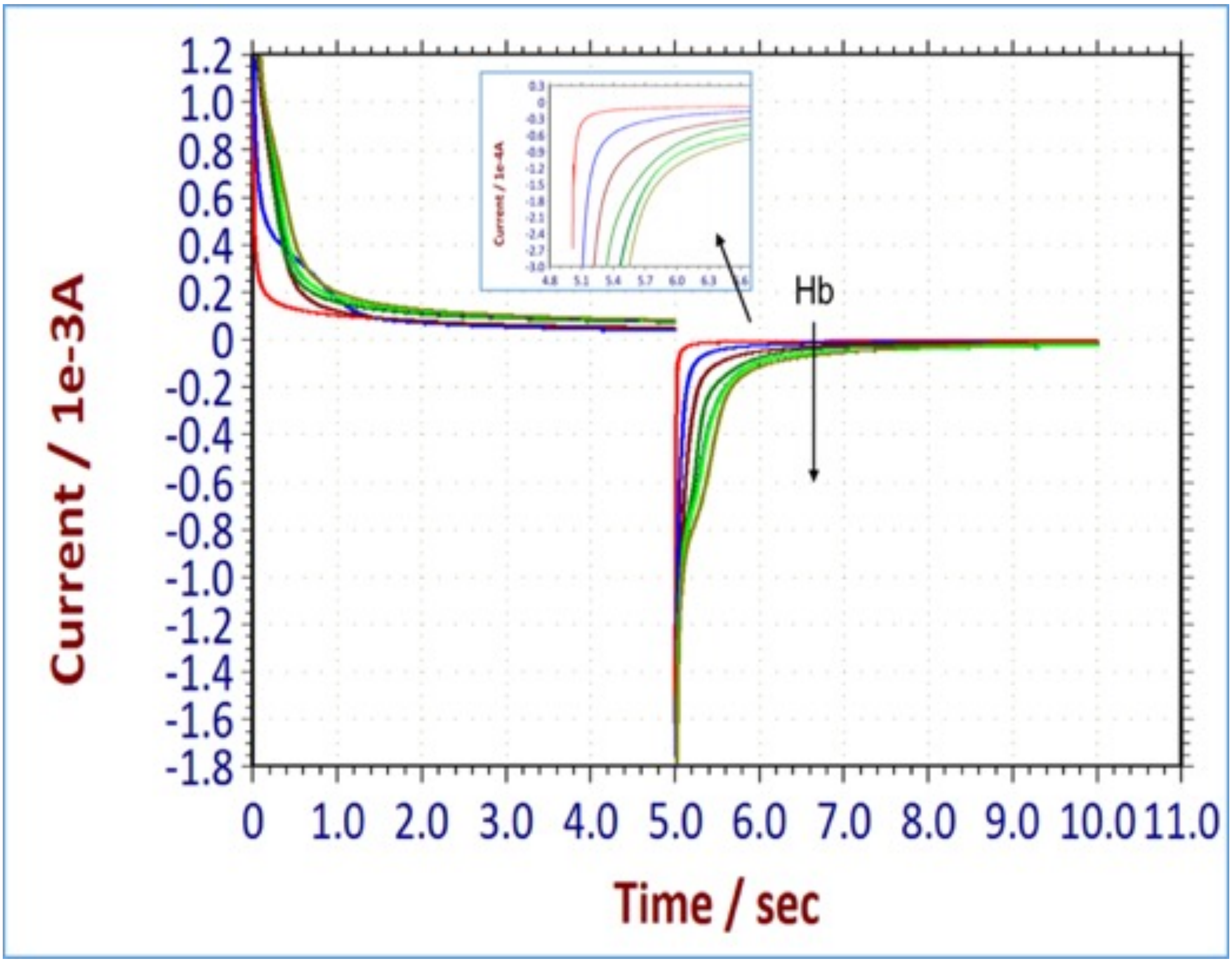
Current / 1e-3A
Time / sec
Hb
Current / 1e-4A

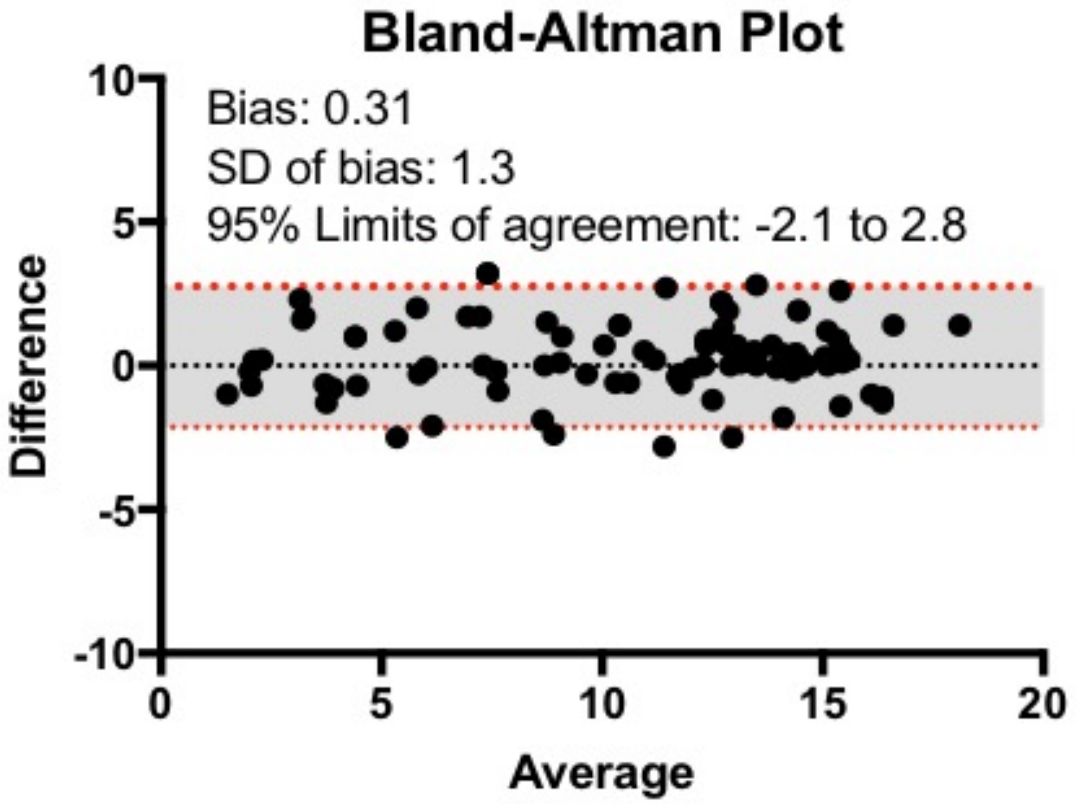
Bland-Altman Plot
Bias: 0.31
SD of bias: 1.3
95% Limits of agreement: -2.1 to 2.8
Difference
Average
10
5
0
-5
-10
0
5
10
15
20
